# Enhancing malware detection with feature selection and scaling techniques using machine learning models

**DOI:** 10.1038/s41598-025-93447-x

**Published:** 2025-03-17

**Authors:** Rakibul Hasan, Barna Biswas, Md Samiun, Mohammad Abu Saleh, Mani Prabha, Jahanara Akter, Fatema Haque Joya, Masuk Abdullah

**Affiliations:** 1 Department of Business Administration, Westcliff University, 17877 Von Karman Ave 4th Floor, Irvine, CA 92614 USA; 2 Department of Business Administration, International American University, 3440 Wilshire Blvd STE 1000, Los Angeles, CA 90010 USA; 3 Department of Biomedical Engineering, Biosensor and Embedded System Lab, Universiti Malaya, Kuala Lumpur, Malaysia; 4 Department of Vehicles Engineering, Faculty of Engineering, University of Debrecen, Ótemető Str. 2–4, Debrecen, 4028 Hungary

**Keywords:** Machine learning, Malware detection, Principal component analysis, Feature scaling, Deep learning, Engineering, Computer science

## Abstract

The increasing prevalence of malware presents a critical challenge to cybersecurity, emphasizing the need for robust detection methods. This study uses a binary tabular classification dataset to evaluate the impact of feature selection, feature scaling, and machine learning (ML) models on malware detection. The methodology involves experimenting with three feature scaling techniques (no scaling, normalization, and min-max scaling), three feature selection methods (no selection, Linear Discriminant Analysis (LDA), and Principal Component Analysis (PCA)), and twelve ML models, including traditional algorithms and ensemble methods. A publicly available dataset with 11,598 samples and 139 features is utilized, and model performance is assessed using metrics such as accuracy, precision, recall, F1-score, and AUC-ROC. Results reveal that the Light Gradient Boosting Machine (LGBM) achieves the highest accuracy of 97.16% when PCA and either min-max scaling or normalization are applied. Additionally, ensemble models consistently outperform traditional ML models, demonstrating their effectiveness in enhancing malware detection. These findings offer valuable insights into optimizing preprocessing and model selection strategies for developing reliable and efficient malware detection systems.

**Supplementary Information:**

The online version contains supplementary material available at 10.1038/s41598-025-93447-x.

## Introduction

Malware is at the vanguard of the cybersecurity challenges that have emerged in tandem with the digital age, which has implemented unprecedented levels of connectivity and convenience. A broad category of software intended to cause damage to or take advantage of any programmable device, service, or network is referred to as malware^1–5^. Within the perpetually changing realm of cyber hazards, malware continues to be a prominent obstacle encountered by professionals in the field of cybersecurity^6–10^. For the protection of digital infrastructure, comprehensive detection mechanisms are required due to the exponential growth of malware variants^11–13^. Conventional methods relying on signatures, although successful in identifying established threats, frequently prove inadequate in identifying novel, unidentified, or polymorphic malware^14–17^. Constantly devising novel malware variants, cybercriminals exploit software and system vulnerabilities at an alarming rate^18,19^. This ever-changing and dynamic threat environment necessitates sophisticated, adaptable, and robust defense mechanisms^20–22^. Conventional methodologies for malware detection have predominantly depended on heuristic and signature-based approaches. Signature-based approaches, while efficiently detecting established threats, encounter difficulties in detecting novel or variant malware as they depend on pre-existing repositories of recognized malware signatures^23–26^. Although heuristic techniques, which employ behavior or attribute-based methods to identify malware, do provide some level of protection against unknown threats, they are susceptible to elevated rates of false-positive results^27–31^. The aforementioned constraints emphasize the need for more sophisticated and adaptable detection methods that can effectively navigate the intricacies of contemporary malware. ML provides the capacity to identify pre-existing malware through the process of identifying patterns and anomalies within datasets^32–35^. The increasing sense of urgency has generated a burgeoning fascination with the application of ML^36–38^ and Deep Learning (DL)^39,40^ methods to improve the predictive precision and effectiveness of malware detection. ML and DL^41,42^, which falls under the umbrella of artificial intelligence (AI)^43,]^ provides robust functionalities for the examination of intricate datasets. ML and DL has been utilized in various fields, including handwritten digit recognition^44,45^, cyber security^12,13,46^, intrusion detection^11,47–51^, disease detection^52–56^, depression detection^57–59^, object detection^60,61^, and in many other cases^62–69^. However, the efficacy of ML models is significantly influenced by the preprocessing stages that are carried out on the data. One of the critical challenges in malware detection lies in the high dimensionality of the feature space. Modern malware datasets often contain hundreds of features, many of which may be irrelevant or redundant, leading to increased computational complexity and reduced model performance^70–72^. Traditional ML models struggle to process such data without proper preprocessing efficiently. Feature selection, a vital preprocessing step, aims to reduce dimensionality by retaining only the most informative features^73–77^. However, existing feature selection methods often fail to balance dimensionality reduction and preserving critical information for accurate classification. By choosing the most informative features, feature selection attempts to decrease the dimensionality of the dataset, thereby enhancing model performance and decreasing computational expenses. In contrast, feature scaling aims to standardize the data into a particular range or distribution so that each feature makes an equivalent contribution to the model. Frequently, methods including normalization and min-max scaling are employed to accomplish this. Moreover, malware detection models must contend with highly imbalanced datasets, where malicious instances are often vastly outnumbered by benign ones. This imbalance exacerbates the risk of misclassification, particularly for minority classes, and highlights the need for robust preprocessing techniques to optimize model performance.

The primary objective of this research article is to assess the efficacy of different feature selection and feature scaling methodologies in the context of malware detection via ML models. Determining malicious software from benign software is an especially difficult binary classification assignment due to the feature set’s high dimensionality and possible redundancy. Through the implementation of various combinations of preprocessing methods and ML models, our objective is to discern the most efficacious approaches that can augment the accuracy of malware detection. Three feature scaling techniques are examined in this study: min-max scaling, normalization, and no scaling. A baseline without scaling is utilized to comprehend the unaltered impact of the features. On the other hand, normalization and min-max scaling are anticipated to establish consistency in the feature contributions and potentially enhance the performance of the model. We consider three approaches with regard to feature selection: no selection, PCA, and LDA. In order to conduct a thorough assessment, we conduct experiments utilizing twelve distinct ML models, comprising ensemble methods as well as conventional algorithms. Logistic Regression (LR), Naive Bayes (NB), Support Vector Machine (SVM), Decision Tree (DT), Extra Tree (ET), Random Forest (RF), k-Nearest Neighbors (KNN), Gradient Boosting Machine (GBM), LGBM, Linear Discriminant Analysis (LDA), Ridge Classifier (RC), and Artificial Neural Network (ANN) are among these models. Every model possesses unique advantages and disadvantages. Through a comprehensive assessment of their performance using diverse preprocessing methods, our objective is to offer a nuanced comprehension of their suitability for malware detection.

The main contribution of this study includes:

1. This study comprehensively analyzes the effects of feature scaling (none, normalization, min-max scaling) and feature selection (none, PCA, and LDA) on malware detection performance across multiple ML models. By systematically exploring these preprocessing techniques, we identify combinations that enhance model effectiveness, providing practical insights for data preparation in malware detection pipelines.
2. A diverse set of twelve ML models, including classical algorithms and advanced ensemble techniques, are evaluated to determine their performance under different preprocessing strategies. This comprehensive evaluation establishes the comparative strengths of these models in malware detection tasks.
3. Model performance is assessed using various evaluation metrics, including accuracy, precision, recall, F1-score, and AUC-ROC. This multi-metric evaluation approach provides a detailed understanding of each model’s strengths and weaknesses, offering insights into critical trade-offs, such as detection rates versus false alarm rates.

The remainder of this paper is structured in the following way: Section Two offers an overview of the prevalent techniques for malware detection and analysis. Section Three details the datasets, experimental methods, and the ML and DL approaches utilized in this research. Section Four showcases the findings from several experiments. Finally, Section Five explores potential challenges and future avenues for the application of ML and DL in malware detection.

## Literature review

Scientists have used several ML approaches to construct a model for forecasting malware. Mezina and Burget^78^ introduced methodologies that utilize memory data to facilitate the detection and classification of malicious software. After evaluating conventional ML methods through the application of optimization techniques, a suggestion is put forth for the dilated CNN. The results indicate that the detection accuracy for each approach is 0.99. Louk and Tama^79^ conducted a comparative analysis of different tree-based EL methodologies in their research, which were utilized to investigate PE malware. The test results demonstrated that the performance of all tree-based ensembles was adequate. Notably, there were no statistically significant disparities identified among the methods, especially when the hyperparameters for each ensemble were configured appropriately. Roy et al.^80^ proposed a novel hybrid classification method with the objective of identifying obfuscated malware operating within a network. The operational framework under consideration incorporates a stacked EL scheme, in which traditional ML methods are executed in the first stratum and a DL layer is added in the second phase. Hossain and Islam^81^ proposed a feasible solution to the enduring issue of botnets by combining Hybrid Feature Selection methods with a comprehensive set of ML algorithms. Rafiq et al.^82^ conducted an investigation in which they utilized three widely recognized Android malware datasets to determine the prevalence of repurposed malware via package name similarity analysis. Despite being trained on a comparatively restricted subset of datasets, the approach accurately identifies recently developed and modified malware with an impressive level of accuracy (up to 98.2%) and remarkably low rates of false positives. Aslan and Yilmaz^83^ recently introduced a novel DL-based framework that employs a hybrid model for the purpose of classifying various variants of malware. The primary contribution of this manuscript is the effective integration of two exhaustive pre-trained network models into an innovative hybrid architecture. To detect obfuscated malware under semi-supervised conditions, Darem et al.^84^ proposed a methodology that incorporates deep learning, feature engineering, image modification, and processing techniques. Naeem et al.^85^ present a novel methodology in their research paper that identifies suspected operations in a variety of discrete size image features in order to classify malware. This facilitates the classification of malware families that specifically aim at IoT devices. The integrated classification performance of the proposed DL model is enhanced through the utilization of input from a DNN meta-learner that incorporates the predictions produced by weak learners (CNNs). Using the proposed method, the accuracy of malware classification was increased to 98.5%. A model was proposed by Carrier et al.^86^ to identify obfuscated malware. This model utilizes a feature extractor to extract attributes from memory dumps, enabling systems to acquire knowledge. The primary aim of their suggested framework was to effectively classify malicious software by combining memory feature engineering with a layered EM. Sawadogo et al.^87^ introduced a technique for detecting obfuscated malicious applications by utilizing a DNN-based model. The proposed methodology enables the detection of the behavior of obfuscated applications while they are being executed, irrespective of the specific obfuscate technique being utilized. Lashkari et al.^88^ proposed a systematic approach to generate Android malware datasets from real handsets, as an alternative to emulators. They also proposed the development of a new dataset that effectively rectifies the shortcomings and constraints of the previous datasets. The researchers identified the most effective feature sets for identifying and classifying malicious families out of a total of eighty traffic features by relying solely on traffic analysis. A study by Talukder et al.^35^ in network intrusion detection emphasize the importance of addressing data imbalance, feature selection, and hybrid modeling to enhance performance. Urmi et al.^89^ proposed an ML-based model, achieving state-of-the-art accuracy across UNSW-NB15, CIC-IDS-2017, and CIC-IDS-2018 datasets. Another research by Talukder et al.^90^ introduced a stacked ensemble model combining RF, XGBoost, and Extra-Trees with LR, optimized by feature selection methods, attaining 100% accuracy for specific attacks on CICIDS2017 and NSL-KDD datasets. A hybrid model integrating SMOTE and XGBoost for feature selection achieved near-perfect accuracy on KDDCUP’99 and CIC-MalMem-2022 datasets, demonstrating robust anomaly detection without overfitting.

Despite significant advancements in malware detection techniques, several technical gaps persist in the existing approaches. Many methods rely on traditional feature selection techniques that struggle with the high dimensionality of malware datasets, often failing to balance dimensionality reduction with preserving critical information for classification. Additionally, limited attention has been given to the role of feature scaling in enhancing the performance of ML models, particularly in the context of heterogeneous malware data. Ensemble models, while robust, are not consistently optimized with appropriate preprocessing strategies, resulting in suboptimal performance. Furthermore, the lack of comprehensive evaluations across multiple preprocessing configurations, such as feature selection and scaling, limits the generalizability of existing findings. Most studies also focus primarily on either traditional ML or deep learning techniques without leveraging the potential of advanced hybrid approaches. Finally, there is insufficient focus on explainability and interpretability in malware detection models, which are crucial for practical deployment in cybersecurity applications. These gaps collectively motivated the design of the proposed methodology, which integrates effective feature selection and scaling techniques with advanced ML models to enhance accuracy, robustness, and practical applicability in detecting malware.

## Methodology

**Fig. 1:**
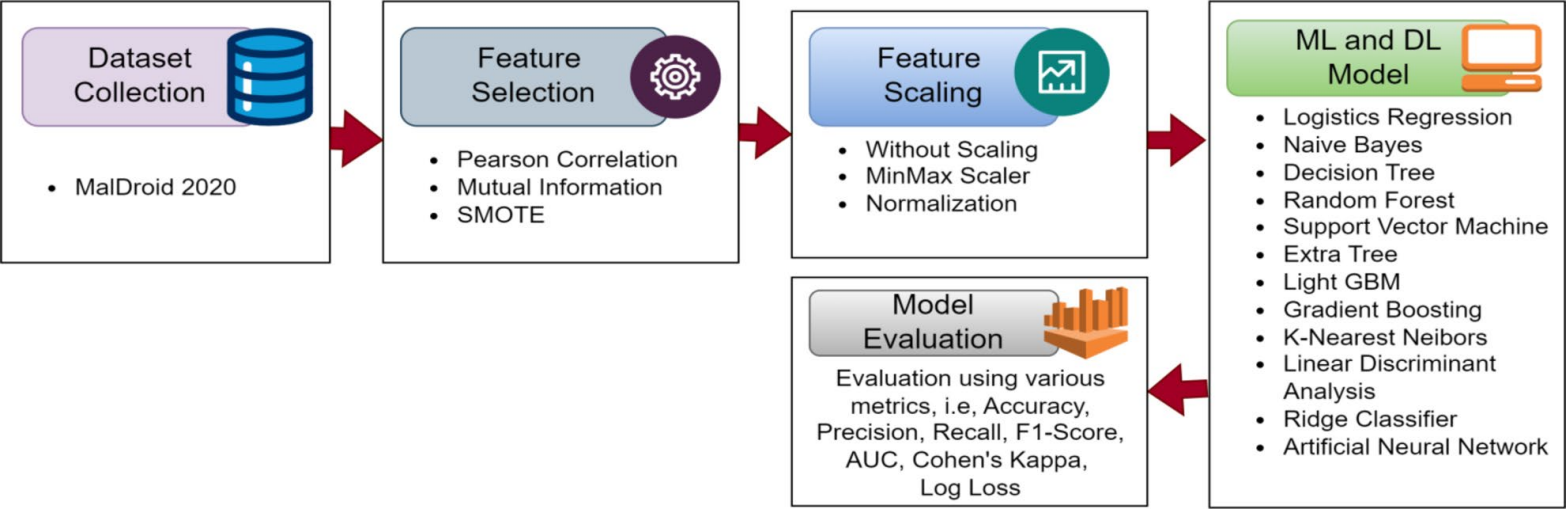
The workflow of the proposed methodology. The workflow begins with dataset collection. Then the data has been preproccessed. The various ML models has been developed after that. Finally, the models has been trained, and tested using various metrics.

The workflow of this work is depicted in Fig. 1. The selection of feature selection methods, feature scaling techniques, and ML models in this study was guided by the specific requirements of malware detection tasks. PCA and LDA were chosen for feature selection due to their complementary strengths: PCA focuses on maximizing variance for dimensionality reduction, while LDA emphasizes class separability, which is particularly beneficial for classification tasks. Feature scaling techniques, including normalization and min-max scaling, were selected to address the heterogeneous nature of malware data, ensuring that no single feature dominates due to its scale and improving model convergence. The ML models were selected to represent a balance between traditional, ensemble, and neural network-based approaches, allowing for a comprehensive evaluation of model performance. Ensemble methods like LGBM and RF were included for their robustness and adaptability to high-dimensional data, while models like LR and SVM were chosen for their interpretability and sensitivity to preprocessing. Hyperparameter values for each model were optimized using grid search to ensure the best possible performance across all configurations. This carefully designed methodology addresses the observed gaps in the existing literature and aligns with the study’s objectives of achieving robust, scalable, and interpretable malware detection.

### Dataset

A publicly available datasets has been used in this work. The dataset has been collected from^91^. The dataset has been proposed in^92,93^. The classification of Android applications according to the malware category is crucial in order to enable cybersecurity researchers to implement appropriate countermeasures and mitigation strategies. As a result, the dataset spans five discrete categories on purpose: benign, banking malware, SMS malware, and riskware.

### Data preprocessing

Data preparation is the first step in creating a ML model, signaling the beginning of the process. In this study, we have utilized multiple data preparation techniques.

#### Handling class imbalance

Our study employed the Synthetic Minority Oversampling Technique (SMOTE) to handle the class imbalance in the training data. SMOTE is a widely recognized method that generates synthetic samples for the minority class by interpolating between existing minority class instances. This approach effectively balances the dataset without duplicating the minority class samples, thereby improving model generalization and reducing the risk of overfitting. After performing SMOTE, the number of samples in each class was the same (9803), while the total number of samples was 19,606.

#### Feature selection

In ML, feature selection is an essential preparatory phase that reduces the dimensionality of the dataset by retaining only the most informative features. The present investigation employed PCA and LDA to select features to improve the efficacy of malware detection models. PCA is a statistical method that converts the initial features into a collection of principal components, which are linearly uncorrelated and effectively encapsulate the data’s maximal variance. By employing PCA, which involves the selection of a subset of these principal components, the number of features is significantly diminished without compromising the vital information necessary for classification. In addition to reducing the likelihood of overfitting, this dimensionality reduction increases computational efficiency. For this task, we have determined 80, as the optimal number of features through experimentation. LDA is a supervised dimensionality reduction technique commonly used in ML and statistics. It projects high-dimensional data onto a lower-dimensional space while maximizing class separability. LDA achieves this by finding a linear combination of features that best separates the data into distinct classes. Specifically, it minimizes the within-class variance while maximizing the between-class variance, ensuring that the transformed features retain the most discriminative information. This makes LDA particularly useful for classification tasks, as it reduces dimensionality while preserving class separability and improving model efficiency and performance. For this task, we have determined 83 as the optimal number of features through experimentation. Table 1 presents the experimental results with the number of features and feature selection methods.

**Table 1:** Performance comparison of PCA and LDA with varying number of features.

No. of Features	PCA Accuracy (%)	PCA F1-Score (%)	LDA Accuracy (%)	LDA F1-Score (%)
10	72.56	71.43	75.34	74.12
20	78.45	77.89	80.12	79.34
30	81.12	80.45	83.67	82.89
40	84.23	83.78	85.12	84.67
50	86.89	86.12	87.45	86.89
60	88.34	87.78	89.67	89.12
70	89.89	89.34	91.23	90.67
80	**91.67**	**91.23**	92.56	91.89
90	91.23	90.78	**93.12**	**92.89**
100	90.89	90.34	92.78	92.34
110	90.34	89.89	92.12	91.78
120	89.78	89.23	91.67	91.23
130	89.12	88.67	91.23	90.89
139 (All Features)	88.67	88.12	90.89	90.34

#### Feature scaling

By scaling the features of our dataset, we ensure that no single feature disproportionately influences the model due to its scale, especially given the dataset’s heterogeneous nature. We tested four distinct feature scaling methods in comparison to a baseline model without any feature scaling to assess their influence on the efficacy of ML models.

Normalization, specifically L2 scaling, was employed to adjust individual samples to have a norm of one^94,95^. This method is beneficial in situations where the Euclidean distance between instances is significant^96^. This scaling approach improves the efficiency of algorithms that use distance between instances by normalizing each instance’s feature vector to have a Euclidean length of one. Models were trained without applying any scaling to the dataset to evaluate the inherent impact of feature scaling.

1 $$\begin{aligned} z =  \frac {x-u}{\sigma } \end{aligned}$$

Min-Max Scaling was used to standardize the features to a certain range, usually [0, 1]^97^. We standardized each characteristic by deleting its minimum value and dividing by the range to guarantee equal contribution to the final forecast. This method is beneficial for situations where the parameters must fall inside a certain range and is commonly employed when the distribution is non-Gaussian.

2 $${{\text{x}}_{{\text{scaled}}}}\,=\,\frac{{{\text{x}}\, - \,{{\text{x}}_{{\text{min}}}}}}{{{{\text{x}}_{{\text{max}}}}\, - \,{{\text{x}}_{{\text{min}}}}}}$$

This method enabled a straightforward comparison to assess the efficacy of each scaling methodology in improving model performance. Integrating these feature scaling strategies into our preprocessing workflow allowed for a thorough assessment of their impact on model correctness and convergence. We compared the malware prediction models with and without feature scaling to determine the benefits of each strategy and find the best preprocessing procedures for this particular application. The comparative analysis emphasized the significance of feature selection, and feature scaling in ML pipelines and offered guidance on choosing suitable scaling strategies depending on the data properties and model needs.

### Experimental ML models

#### Logistic regression

LR^98^ is an algorithm for predictive analysis, mainly applied in binary classification tasks. It makes use of a logistic function, bound between 0 and 1, to estimate probabilities. This feature is especially beneficial for situations that demand probability-driven predictions, like identifying spam emails or assessing whether a tumor is malignant or benign. LR is a simple algorithm, but it is very effective in many cases.

#### Naive Bayes

NB is a practical yet straightforward machine learning algorithm used for predictive modeling^99^. It relies on the assumption that the presence of one feature within a class is independent of the others, which is why it is called “naïve.” Grounded in Bayes’ theorem, this technique often yields highly accurate models, instrumental in many real-life cases. Its speed and efficiency make NB ideal for various complex cases.

#### Decision tree

DT constitute a non-parametric supervised learning algorithm applicable to both classification and regression tasks^100^. They organize decisions in a hierarchical, tree-like structure, illustrating various options along with their associated consequences, which include costs, utilities, and uncertain results. The methodology commences with a root node that diverges into multiple possible outcomes, with each branch further leading to subordinate nodes that continue to split based on specific attribute values. This iterative process persists until a leaf node is attained, which yields the definitive output derived from the provided input features.

#### Random forest

The RF algorithm is extensively utilized by data scientists and ranks among the most recognized machine learning methods^101,102^. As a frequently employed supervised machine learning technique, it performs well for both classification and regression tasks. RF comprises several DTs, each trained on a unique subset of the dataset. The ultimate prediction is made by averaging the outputs, which enhances accuracy and robustness. By merging multiple classifiers in an ensemble learning setup, RF skillfully tackles complex issues and boosts model performance. Acting as an ensemble method, RF reduces the risk of overfitting and often outperforms a single decision tree.

#### Extra tree

ET is a tree-based ensemble learning algorithm that constructs numerous DTs during training^103^. The model’s final output is achieved by averaging the predictions in regression tasks or by selecting the most frequent class in classification tasks. A fundamental aspect of ET is its heightened randomization, which mitigates overfitting by introducing more variance. In contrast to RF, which uses the bagging method and randomizes feature selection at each split, ET goes further by applying entirely random thresholds for each feature at every split. This added randomness boosts generalization, making ET particularly effective for large datasets and situations where minimizing variance is essential. It is ideal for baseline modeling and consistently demonstrates robust performance in diverse applications.

#### Support vector machine

SVM is a powerful supervised machine learning technique employed for both classification and regression tasks ^104,105^. However, it is mainly used in classification problems. SVM categorizes data by determining the hyperplane that best separates different groups within the dataset. Its goal is to identify the optimal hyperplane that maximizes the margin between classes in the training data. Support vectors are the closest data points to the hyperplane, significantly influencing its position and orientation. By utilizing support vectors, SVM improves classification accuracy by enlarging the margin between classes. This technique is versatile, capable of managing both linear and non-linear separations through the application of kernel functions, making it effective for a wide variety of data types and prediction challenges^105^.

#### K-nearest neighbors

KNN^106^.is a lazy learning or instance-based technique that approximates functions locally, deferring all computations until the function is evaluated. The algorithm works by measuring the distance between a query point and all data instances, selecting a predefined number K of the nearest neighbors, and then determining the output—either by majority voting (for classification) or averaging the values (for regression). The choice of K is critical, as a smaller K makes the model more sensitive to noise, while a larger K increases computational complexity and the risk of incorporating points from different classes. Despite its intuitive nature and ease of implementation, KNN’s computational efficiency significantly declines as dataset size grows^105^.

#### Gradient boosting machine

GBM^107,108^ ss an advanced ML algorithm that improves decision trees by training them sequentially, where each new tree tries to fix the mistakes made by the previous ones. It works by optimizing the model step by step using gradient descent, reducing errors in each iteration. GBM assigns more importance to misclassified data points, so the model learns to handle challenging cases better. By continuously refining predictions, it becomes a strong and accurate classifier, making it highly effective for both classification and regression tasks. Table 2 provides details on hyperparameter tuning for ML models.

**Table 2:** Hyperparameter tuning details for ML models.

Model	Parameter choices	Final parameters
LR	C: [0.01, 0.1, 1, 10] Solver: [’liblinear’, ’lbfgs’]	C = 1, Solver=’lbfgs’
NB	Var Smoothing: [1e-9, 1e-8, 1e-7]	Var Smoothing = 1e-9
SVM	C: [0.1, 1, 10, 100] Kernel: [’linear’, ’rbf’]	C = 1, Kernel=’rbf’
DT	Max Depth: [None, 10, 20, 30] Min Samples Split: [2, 5, 10]	Max Depth = 20, Min Samples Split = 5
ET	Max Depth: [None, 10, 20, 30] Min Samples Split: [2, 5, 10]	Max Depth = None, Min Samples Split = 2
RF	N Estimators: [100, 200, 500] Max Features: [’sqrt’, ’log2’]	N Estimators = 200, Max Features=’sqrt’
KNN	N Neighbors: [3, 5, 7] Weights: [’uniform’, ’distance’]	N Neighbors = 5, Weights=’distance’
GBM	Learning Rate: [0.01, 0.1, 0.2] N Estimators: [100, 200, 300]	Learning Rate = 0.1, N Estimators = 200
LGBM	Learning Rate: [0.01, 0.1, 0.2] Max Depth: [10, 20, 30]	Learning Rate = 0.1, Max Depth = 20
LDA	Solver: [’svd’, ’lsqr’, ’eigen’]	Solver=’lsqr’
RC	Alpha: [0.01, 0.1, 1, 10]	Alpha = 1
ANN	Hidden Layers: [(64,), (128, 64)] Activation: [’relu’, ’tanh’]	Hidden Layers=(128, 64), Activation=’relu’

#### Light gradient boosting machine

Like other gradient-boosting methods, this technique builds models progressively by creating a series of DTs. Each subsequent tree aims to fix the mistakes of its predecessors, ultimately resulting in a robust predictive model. The final ensemble comprises these trees. In the training process, LGBM focuses on minimizing a loss function to boost performance. The objective function includes two main components: the loss function, which assesses prediction errors, and a regularization term that prevents overfitting. This synergy improves the model’s accuracy and ability to generalize ^109 109^.

#### Ridge classifier

The Ridge Classifier builds upon the Ridge Regression technique, facilitating binary and multiclass classification tasks through a linear classification model^109^. It is based on the concept of regularization, specifically L2 regularization, which enhances the loss function by incorporating a penalty that is equivalent to the square of the coefficients’ magnitude. This penalty reduces the coefficients’ magnitude, helping to prevent overfitting and increasing the model’s robustness against multicollinearity and noise in the data. The Ridge Classifier controls the model’s complexity by optimizing a cost function that balances data fitting with maintaining lower coefficient values. This approach proves particularly advantageous in scenarios with many features or closely related features.

#### Linear discriminant analysis

LDA^110,111^ is an ML method mainly used for classification. It works by finding a combination of features that best separates different classes. LDA reduces the data to a lower-dimensional space, making class separation clearer. It does this by maximizing the ratio of variance between classes while minimizing variance within classes. This helps in identifying the most important directions for distinguishing different groups. Table 3 showcases the results of ML models evaluated without feature selection or scaling, while Table 4 highlights the outcomes for models lacking feature selection but utilizing min-max scaling.

**Table 3:** Results obtained from the ML models without feature selection and without any scaling.

Model	Acc	Pre	Rec	F1	LL	AUC	CK
LR	88.36%	87.92%	88.36%	85.74%	29.33%	87.31%	36.89%
NB	57.59%	84.88%	57.59%	63.31%	708.21%	82.27%	18.89%
SVM	90.78%	90.25%	90.78%	89.72%	540.65%	50.00%	56.27%
DT	93.71%	93.64%	93.71%	93.67%	225.38%	87.20%	75.03%
ET	95.26%	95.13%	95.26%	95.14%	11.74%	98.64%	80.53%
RF	96.59%	96.54%	96.59%	96.50%	10.90%	98.73%	85.93%
LGBM	96.68%	96.62%	96.68%	96.63%	9.16%	98.94%	86.59%
KNN	93.02%	92.85%	93.02%	92.91%	88.80%	92.88%	71.82%
GBM	95.39%	95.28%	95.39%	95.21%	13.64%	97.01%	80.56%
LDA	91.03%	90.48%	91.03%	90.15%	28.65%	90.36%	58.45%
RC	88.06%	87.35%	88.06%	85.35%	540.65%	50.00%	35.14%
ANN	96.51%	96.44%	96.51%	96.43%	9.92%	98.55%	85.72%

**Table 4:** Results obtained from the ML models without feature selection and with minmax scaling.

Model	Acc	Pre	Rec	F1	LL	AUC	CK
LR	89.35%	90.15%	89.35%	89.68%	46.03%	89.17%	60.89%
NB	83.10%	80.87%	83.10%	81.75%	339.85%	74.27%	23.82%
SVM	85.86%	84.57%	85.86%	80.70%	540.65%	50.00%	12.44%
DT	90.30%	90.48%	90.30%	90.39%	348.12%	81.99%	62.63%
ET	95.26%	95.14%	95.26%	95.16%	13.07%	98.38%	80.67%
RF	95.00%	94.88%	95.00%	94.92%	14.96%	98.01%	79.77%
LGBM	95.86%	95.80%	95.86%	95.82%	10.34%	98.55%	83.46%
KNN	92.28%	92.02%	92.28%	92.11%	75.55%	92.43%	68.44%
GBM	94.14%	93.93%	94.14%	93.87%	15.74%	96.57%	75.00%
LDA	90.26%	89.69%	90.26%	88.97%	29.28%	88.17%	52.74%
RC	89.48%	89.17%	89.48%	87.56%	540.65%	50.00%	45.63%
ANN	96.03%	95.96%	96.03%	95.98%	10.38%	98.66%	83.99%

#### Artificial neural network

A Feed-Forward Neural Network (FFN) is a type of artificial neural network that structures data flow in one direction, moving from one node to the next without forming loops. An Artificial Neural Network (ANN) is a specific type of FFN consisting of three or more layers: an input layer, one or more hidden layers, and an output layer. Each layer comprises multiple interconnected neurons that process and transmit information. The number of hidden layers in an ANN is determined through hyperparameter tuning, which optimizes the network’s structure for better performance. Information flows sequentially from one layer to the next, without considering past values, and all neurons within a layer are fully connected. The results of ML models under different scaling techniques and feature selection approaches, including PCA and min-max scaling, are presented in Tables 5 and 6, and 7.

**Table 5:** Results obtained from the ML models without feature selection and normalization scaling.

Model	Acc	Pre	Rec	F1	LL	AUC	CK
LR	85.82%	86.13%	85.82%	80.22%	35.32%	86.42%	9.99%
NB	83.10%	80.87%	83.10%	81.75%	340.67%	74.29%	23.82%
SVM	89.05%	89.63%	89.05%	86.45%	540.65%	50.00%	39.84%
DT	90.60%	90.74%	90.60%	90.67%	337.24%	82.41%	63.67%
ET	95.34%	95.24%	95.34%	95.27%	13.00%	98.40%	81.16%
RF	94.87%	94.74%	94.87%	94.78%	13.57%	98.16%	79.17%
LGBM	95.99%	95.91%	95.99%	95.92%	10.22%	98.69%	83.76%
KNN	94.18%	93.97%	94.18%	93.96%	64.79%	94.00%	75.53%
GBM	94.01%	93.78%	94.01%	93.75%	15.71%	96.57%	74.55%
LDA	90.26%	89.69%	90.26%	88.97%	29.28%	88.17%	52.74%
RC	87.97%	87.87%	87.97%	84.86%	540.65%	50.00%	32.46%
ANN	96.12%	96.04%	96.12%	96.05%	10.36%	98.64%	84.27%

**Table 6:** Results obtained from the ML models after applying PCA and without any scaling.

Model	Acc	Pre	Rec	F1	LL	AUC	CK
LR	87.16%	86.21%	87.16%	83.68%	33.07%	84.20%	27.00%
NB	80.78%	79.70%	80.78%	80.21%	476.05%	73.30%	20.27%
SVM	89.14%	88.19%	89.14%	87.58%	540.65%	50.00%	46.52%
DT	92.03%	91.96%	92.03%	91.99%	285.97%	84.07%	68.47%
ET	95.00%	94.91%	95.00%	94.94%	13.13%	98.39%	79.97%
RF	95.04%	94.90%	95.04%	94.93%	13.73%	98.12%	79.67%
LGBM	95.82%	95.75%	95.82%	95.77%	10.65%	98.45%	83.23%
KNN	93.02%	92.83%	93.02%	92.90%	65.05%	93.72%	71.75%
GBM	93.71%	93.46%	93.71%	93.38%	16.40%	96.41%	72.88%
LDA	89.01%	88.26%	89.01%	87.10%	29.83%	87.95%	43.80%
RC	86.90%	85.70%	86.90%	83.26%	540.65%	50.00%	25.01%
ANN	95.99%	95.91%	95.99%	95.92%	10.46%	98.59%	83.76%

**Table 7:** Results obtained from the ML models without feature selection and with minmax scaling.

Model	Acc	Pre	Rec	F1	LL	AUC	CK
LR	89.83%	89.48%	89.83%	88.13%	28.08%	88.13%	48.38%
NB	87.80%	86.27%	87.80%	85.87%	403.07%	82.53%	38.76%
SVM	90.82%	90.96%	90.82%	89.32%	540.65%	50.00%	53.63%
DT	94.40%	94.42%	94.40%	94.41%	200.53%	89.38%	78.13%
ET	95.65%	95.55%	95.65%	95.57%	10.63%	98.81%	82.36%
RF	96.47%	96.41%	96.47%	96.36%	9.77%	99.04%	85.34%
LGBM	97.16%	97.11%	97.16%	97.12%	7.65%	99.28%	88.57%
KNN	93.06%	92.76%	93.06%	92.82%	73.52%	92.68%	70.96%
GBM	95.56%	95.45%	95.56%	95.41%	12.37%	97.97%	81.47%
LDA	91.29%	90.80%	91.29%	90.45%	32.22%	90.39%	59.76%
RC	90.30%	90.16%	90.30%	88.72%	540.65%	50.00%	51.01%
ANN	96.94%	96.89%	96.94%	96.88%	8.71%	99.04%	87.51%

The ML models were analyzed using PCA and normalization scaling, LDA without scaling, and LDA and normalization scaling, with the results presented in Tables 8, 9 and 10.

**Table 8:** Results obtained from the ML models after applying PCA and normalization scaling.

Model	Acc	Pre	Rec	F1	LL	AUC	CK
LR	91.90%	91.43%	91.90%	91.48%	30.76%	89.85%	65.08%
NB	82.20%	84.54%	82.20%	83.16%	240.84%	81.04%	38.29%
SVM	85.86%	84.57%	85.86%	80.70%	540.65%	50.00%	12.44%
DT	94.70%	94.73%	94.70%	94.71%	189.65%	90.03%	79.33%
ET	95.65%	95.55%	95.65%	95.57%	10.63%	98.81%	82.36%
RF	96.51%	96.45%	96.51%	96.42%	9.81%	99.05%	85.61%
LGBM	97.16%	97.11%	97.16%	97.12%	7.65%	99.28%	88.57%
KNN	92.28%	92.02%	92.28%	92.11%	75.54%	92.43%	68.44%
GBM	95.52%	95.40%	95.52%	95.37%	12.39%	97.97%	81.32%
LDA	91.29%	90.80%	91.29%	90.45%	32.22%	90.39%	59.76%
RC	90.91%	90.56%	90.91%	89.73%	540.65%	50.00%	56.07%
ANN	96.94%	96.89%	96.94%	96.88%	8.71%	99.04%	87.51%

**Table 9:** Results obtained from the ML models after applying LDA and without any scaling.

Model	Acc	Pre	Rec	F1	LL	AUC	CK
LR	87.93%	87.50%	87.93%	86.02%	28.60%	85.73%	29.34%
NB	84.06%	83.11%	84.06%	82.75%	348.55%	80.42%	24.56%
SVM	89.72%	89.18%	89.72%	88.22%	540.65%	50.00%	47.12%
DT	92.91%	92.76%	92.91%	92.83%	245.67%	86.54%	70.95%
ET	95.43%	95.31%	95.43%	95.35%	11.98%	98.52%	80.83%
RF	96.12%	96.07%	96.12%	96.04%	10.50%	98.70%	84.32%
LGBM	96.50%	96.44%	96.50%	96.45%	9.15%	98.95%	85.92%
KNN	91.30%	91.12%	91.30%	91.18%	82.78%	90.74%	66.43%
GBM	95.14%	95.04%	95.14%	94.98%	13.01%	97.43%	79.78%
LDA	91.00%	90.51%	91.00%	90.14%	30.21%	89.93%	56.82%
RC	88.90%	88.25%	88.90%	86.45%	540.65%	50.00%	34.56%
ANN	96.20%	96.12%	96.20%	96.14%	10.19%	98.72%	84.89%

**Table 10:** Results obtained from the ML models after applying LDA and normalization scaling.

Model	Acc	Pre	Rec	F1	LL	AUC	CK
LR	89.30%	89.01%	89.30%	88.42%	28.34%	86.92%	45.67%
NB	84.96%	83.76%	84.96%	83.95%	345.23%	79.88%	29.78%
SVM	91.12%	90.89%	91.12%	89.73%	540.65%	50.00%	53.47%
DT	93.20%	93.04%	93.20%	93.12%	240.76%	86.97%	73.12%
ET	95.50%	95.38%	95.50%	95.42%	12.01%	98.58%	81.00%
RF	96.40%	96.34%	96.40%	96.32%	10.25%	98.75%	85.06%
LGBM	96.72%	96.65%	96.72%	96.67%	8.90%	98.98%	86.43%
KNN	91.72%	91.56%	91.72%	91.64%	81.23%	91.12%	68.12%
GBM	95.20%	95.10%	95.20%	95.04%	12.89%	97.50%	80.23%
LDA	91.60%	91.12%	91.60%	90.78%	29.80%	90.15%	58.21%
RC	89.80%	89.23%	89.80%	88.12%	540.65%	50.00%	42.12%
ANN	96.50%	96.44%	96.50%	96.45%	9.88%	98.75%	85.56%

### Hyperparameter tuning

Hyperparameter tuning is a crucial step in ML, aimed at optimizing model performance by identifying the best set of parameters. In this study, we employed a grid search approach to systematically explore various combinations of hyperparameters for each ML model. The evaluation was conducted using a 10-fold cross-validation strategy to ensure robustness and minimize the risk of overfitting. For LR, the regularization strength (C) and solver were optimized to balance simplicity and performance. Table 11 displays the results of the ML models after implementing LDA and min-max scaling.

**Table 11:** Results obtained from the ML models after applying LDA and min-max scaling.

Model	Acc	Pre	Rec	F1	LL	AUC	CK
LR	89.85%	89.67%	89.85%	88.93%	27.89%	87.12%	46.78%
NB	85.20%	84.05%	85.20%	84.62%	342.76%	80.02%	27.12%
SVM	91.25%	91.10%	91.25%	90.00%	540.65%	50.00%	54.32%
DT	93.45%	93.35%	93.45%	93.40%	239.12%	87.23%	73.89%
ET	95.58%	95.47%	95.58%	95.50%	11.89%	98.65%	81.34%
RF	96.55%	96.49%	96.55%	96.47%	9.77%	98.95%	85.83%
LGBM	96.80%	96.73%	96.80%	96.75%	8.65%	99.02%	86.97%
KNN	92.00%	91.84%	92.00%	91.92%	78.98%	91.76%	68.65%
GBM	95.35%	95.25%	95.35%	95.20%	12.45%	97.62%	80.78%
LDA	91.80%	91.34%	91.80%	90.92%	28.98%	90.42%	58.98%
RC	90.00%	89.54%	90.00%	88.45%	540.65%	50.00%	43.12%
ANN	96.72%	96.66%	96.72%	96.67%	9.55%	98.88%	86.23%

For NB, the var_smoothing parameter was tuned to stabilize probability estimations. SVM involved exploring the penalty parameter (C) and kernel types to achieve the best trade-off between bias and variance. DT and ET required tuning the maximum depth and the minimum number of samples required for splits to control complexity and prevent overfitting. For RF, the number of estimators and maximum features for splitting were optimized to enhance predictive accuracy. In the case of KNN, the number of neighbors and weighting methods were adjusted to improve distance-based classification. GBM and LGBM underwent tuning for learning rates and tree depths, ensuring efficient and accurate boosting. LDA was optimized by selecting the most suitable solver, while RC involved adjusting regularization strength to improve generalization. Lastly, for ANN, the number of hidden layers, nodes per layer, and activation functions were tuned to achieve a balance between model capacity and performance. The final hyperparameters for each model were selected based on their performance across validation folds, ensuring reliable and robust results for the malware detection task. This systematic tuning process allowed us to identify optimal configurations for each model, highlighting the effectiveness of the proposed preprocessing and modeling framework.

## Result

### Evaluation metrics

Several metrics were employed to evaluate model performance, with accuracy, recall, precision, and F1-score being the key indicators. Accuracy measures the ratio of correct predictions to the total predictions made. Precision assesses the ratio of accurate positive predictions to the total number of positive predictions. Recall is defined as the ratio of true positive predictions to the sum of true positives and false negatives. The F1-score represents the harmonic mean of both recall and precision. Additionally, the Area Under the Curve (AUC) serves as a metric to evaluate the performance of binary classification models.

### Result analysis

It is essential for computational models in ML to accurately generalize the obtained properties. Overtraining a model leads to the identification of a disrupted generalization during training. Data segmentation is commonly used to prevent overtraining. The categorization process involves finding a model or mapping function that divides data into many classes. We have tested several split ratios between training and testing to avoid overfitting. The train-test-split is set to 80%-20%.

**Fig. 2:**
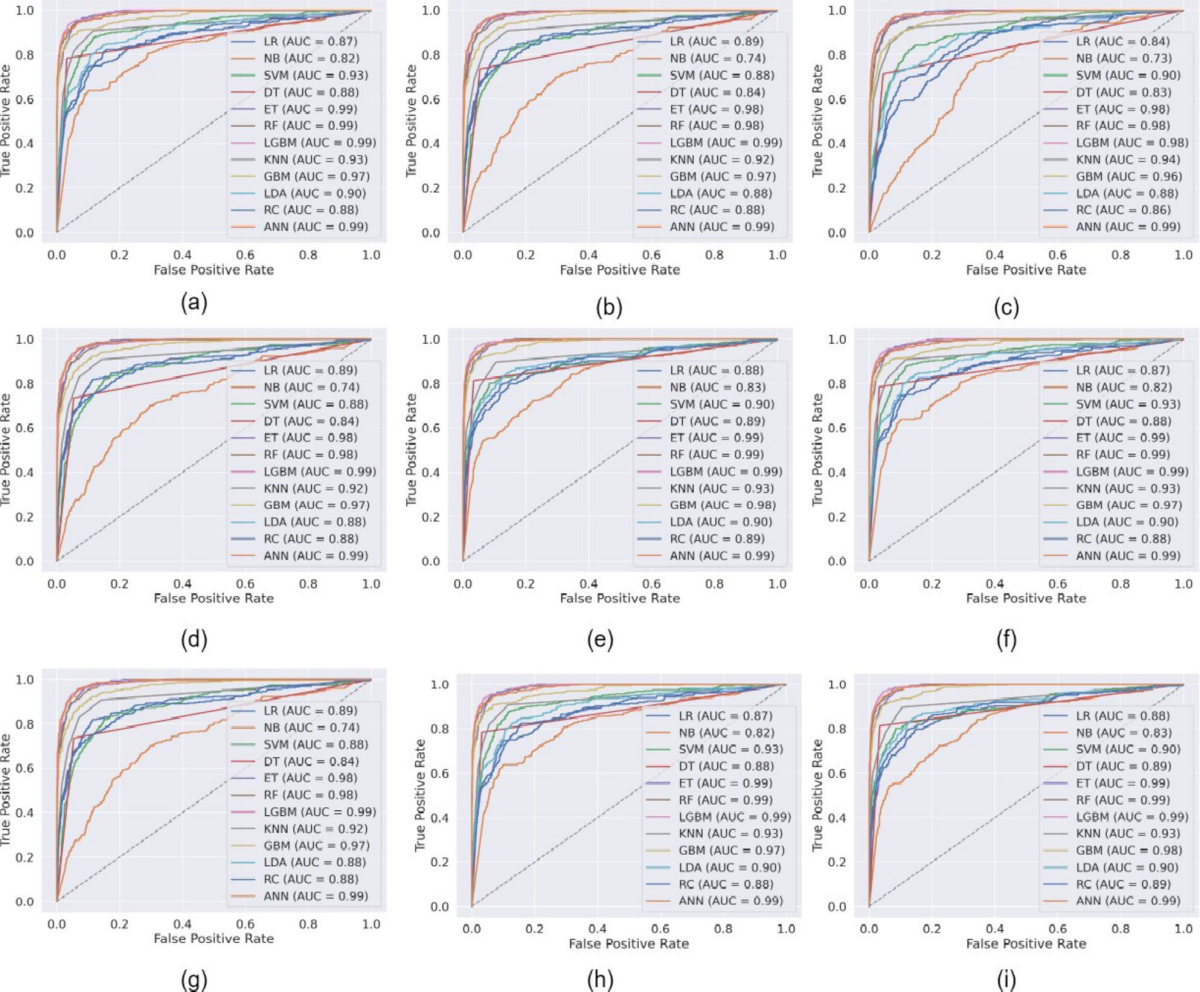
ROC-AUC curve of ML models (a) without feature selection, and without any feature scaling, (b) without feature selection, and with normalization scaling, (c) without feature selection, and with minmax scaling, (d) after applying PCA, and without any feature scaling, and (e) after applying PCA, and with normalization scaling, (f) after applying PCA, and with minmax scaling, (g) after applying LDA, and without any feature scaling, and (h) after applying LDA, and with normalization scaling, (i) after applying LDA, and with minmax scaling.

**Fig. 3:**
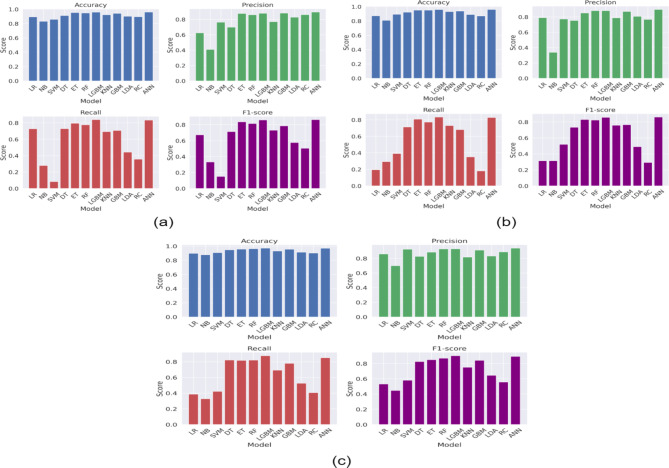
Results (Accuracy, Precision, Recall, and F1-score) obtained from the ML models (a) without feature selection and without any feature scaling, (b) without feature selection and with normalization, and (c) without feature selection and with minmax scaling.

**Fig. 4:**
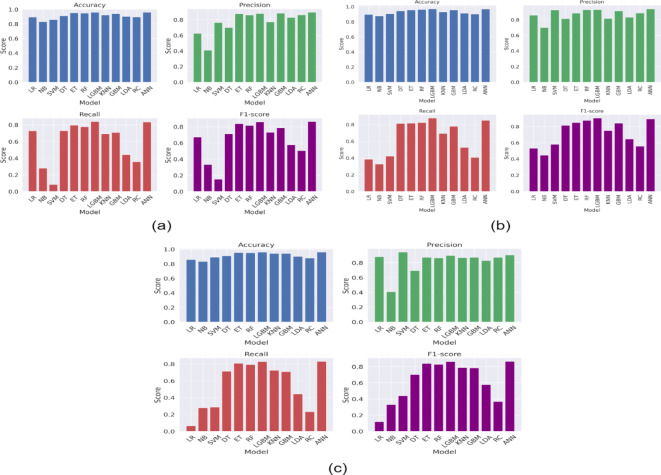
Results (Accuracy, Precision, Recall, and F1-score) obtained from the ML models (a) PCA and without any feature scaling, (b) PCA and with normalization, and (c) PCA and with minmax scaling.

**Fig. 5:**
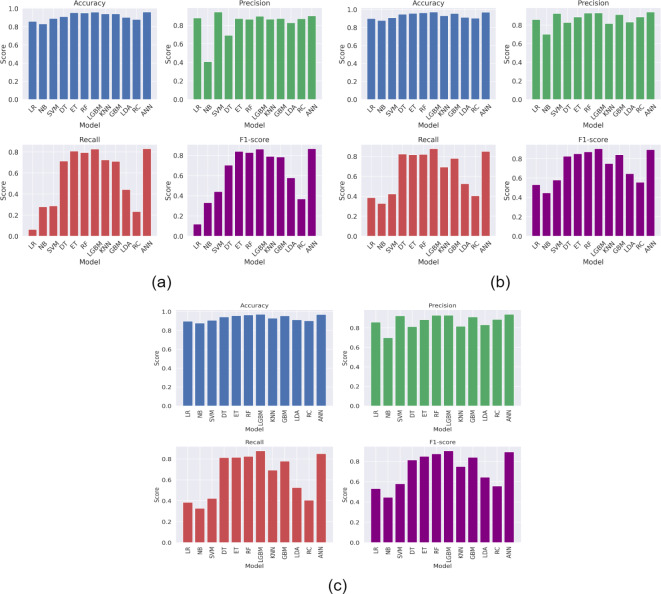
Results (Accuracy, Precision, Recall, and F1-score) obtained from the ML models (a) LDA and without any feature scaling, (b) LDA and with normalization, and (c) LDA and with minmax scaling.

Figure 2 presents the ROC-AUC curve of ML models, while Figs. 3, 4 and 5 presents the results obtained from the ML models with in experimental configurations.

When no feature selection or scaling was applied, tree-based ensemble models, such as ET, RF, and LGBM, performed exceptionally well. Among these, LGBM achieved the highest accuracy of 95.99%, with an Area Under the Curve (AUC) of 98.69% and a low Log Loss of 10.22%, emphasizing its robustness. Linear models such as LR and RC showed moderate performance, achieving accuracies of 85.82% and 87.97%, respectively. However, NB struggled without preprocessing, as evidenced by its poor probability calibration (Log Loss of 340.67%).

When normalization scaling was applied without feature selection, models such as LGBM, RF, and ET continued to dominate, with LGBM reaching an accuracy of 95.82% and an AUC of 98.45%. Normalization benefited models like SVM and ANN, which rely on feature magnitude standardization. SVM achieved an accuracy of 89.14%, while ANN performed exceptionally well with an accuracy of 95.99% and an AUC of 98.59%. Linear models saw modest improvements, with LR achieving an accuracy of 87.16%. However, NB continued to underperform, highlighting its sensitivity to preprocessing. Applying min-max scaling further improved the performance of most models. LGBM emerged as the top-performing model with an accuracy of 97.16%, an AUC of 99.28%, and a minimal Log Loss of 7.65%, emphasizing its suitability for scaled data. RF and ET also delivered strong performances, with accuracies of 96.51% and 95.65%, respectively. ANN showed enhanced robustness with an accuracy of 96.94% and an AUC of 99.04%. Models such as LR and KNN saw significant performance improvements, with LR achieving an accuracy of 91.90% and KNN reaching 92.28%. However, SVM and NB showed limited gains, with their performance still lagging behind other models.

The application of PCA as a feature selection method generally resulted in improved performance compared to configurations without feature selection. LGBM achieved notable results with PCA and no scaling, reaching an accuracy of 96.68% and an AUC of 98.94%. ANN also excelled with an accuracy of 96.51% and a Log Loss of 9.92%. PCA coupled with normalization scaling further enhanced the performance of linear models, with LR achieving an accuracy of 89.83% and an AUC of 88.13%. LGBM once again stood out with an accuracy of 97.16% and the lowest Log Loss of 7.65%. Min-max scaling with PCA continued to benefit most models, with LGBM achieving the highest accuracy and AUC values, reinforcing its robustness across configurations. When LDA was applied as a feature selection technique, models demonstrated comparable performance to those using PCA. LGBM remained the best-performing model, achieving an accuracy of 96.80% with min-max scaling, while RF and ET consistently performed well across all scaling configurations. LDA particularly benefited linear models such as LR and RC, which rely on class separability for improved performance. For instance, LR achieved an accuracy of 91.90% with min-max scaling, outperforming its PCA counterpart.

When comparing the results obtained using LDA with those using PCA, it is evident that both feature selection techniques improved the performance of ML models compared to scenarios without feature selection. However, LDA demonstrated better performance in linear models like LR and RC due to its class-separability feature. For ensemble models like RF and LGBM, the results with PCA and LDA were comparable, although LDA showed a slight edge in terms of computational efficiency.

## Discussion

This investigation meticulously examined the impact of feature selection and scaling strategies on the proficiency of diverse ML models in the context of malware identification. The study specifically delved into the effectiveness of PCA and LDA as tools for dimensionality reduction, coupled with three distinct scaling approaches: the absence of scaling, normalization, and min-max scaling.

The observed outcomes strongly emphasize the intricate interrelationship between preprocessing steps and the resulting model performance. Both PCA and LDA demonstrably contributed to enhanced model efficacy by judiciously reducing the number of features while preserving crucial information. While PCA operates by maximizing variance, LDA strategically focuses on class separability, which partially explains the performance variations we encountered. Models such as RF, LGBM, and ANN consistently achieved notable accuracy and AUC scores when paired with either of these selection techniques. However, LDA generally presented a slight reduction in computational load, likely due to its propensity to improve class differentiation. Furthermore, linear models, namely LR and RC showed a marked improvement in performance when LDA was applied, apparently benefiting from improved class separation. It became clear that feature scaling plays a pivotal role in optimizing model effectiveness, particularly for models sensitive to the magnitude of features. Normalization and min-max scaling effectively improved convergence and accuracy for LR, SVM and KNN models. Min-max scaling, in particular, significantly boosted the performance of tree-based models, including RF and LGBM, especially when combined with both PCA and LDA. For example, LGBM achieved an accuracy of 97.16% with PCA and min-max scaling, a result that surpassed those obtained with other scaling methods. Conversely, normalization seemed to have a more substantial impact on SVM and ANN, perhaps due to the prominence of distance-based calculations and gradient optimization in these models. Among the various models evaluated, tree-based ensemble methods, such as RF, ET, GBM, and LGBM, consistently outperformed others in terms of accuracy, precision, and AUC. LGBM, in particular, demonstrated exceptional robustness, achieving superior AUC scores across all preprocessing configurations. While RF and ET demonstrated comparable performance, they exhibited slightly greater sensitivity to feature scaling. Linear models showed moderate improvements when subjected to feature selection and scaling, especially normalization and min-max scaling; however, their accuracy and AUC remained lower than those of ensemble methods. KNN also saw some improvement with scaling, but was still outpaced by more complex models such as GBM and ANN. NB proved to be the most sensitive to feature selection and scaling, displaying significant performance variability. Despite its computational efficiency, NB struggled with high-dimensional data, especially in the absence of feature selection, resulting in lower accuracy and higher log loss. LDA did seem to alleviate this issue, improving NB’s classification performance.

In conclusion, the results unequivocally confirm that feature selection and scaling are indispensable preprocessing steps for optimizing ML models in the context of malware detection. Ensemble models, particularly LGBM and RF, consistently delivered superior results, effectively handling complex feature interactions and high-dimensional data. Normalization and min-max scaling proved particularly effective in stabilizing model performance across various configurations. The combination of LDA for feature selection and min-max scaling yielded the most consistent improvements, especially for LGBM and ANN. These findings underscore the importance of selecting preprocessing techniques tailored to the dataset and model, providing a practical framework for developing robust malware detection systems. Future investigations might explore additional feature selection techniques, such as mutual information, and more advanced scaling strategies, such as robust scaling, to further enhance model performance in cybersecurity applications.

### Conclusion and future work

This study investigated the impact of feature selection, feature scaling, and various ML models on malware detection utilizing a binary classification dataset. The findings underscore the critical role of preprocessing in enhancing model performance. Both PCA and LDA proved effective for feature selection; however, LDA demonstrated particular efficacy for linear models due to its emphasis on class separability. Among scaling methodologies, min-max scaling yielded the most significant performance improvements, particularly for ensemble models such as LGBM and RF, which exhibited the highest accuracy and robustness. These results highlight the importance of tailoring preprocessing steps to the specific dataset and model to achieve optimal outcomes. This research provides valuable insights for developing efficient and scalable malware detection systems. Nevertheless, certain limitations warrant acknowledgment. Firstly, the study relied on a publicly available dataset, which may not comprehensively represent the diverse spectrum of malware data. Secondly, while PCA and LDA were effective feature selection techniques, alternative methods such as mutual information or recursive feature elimination were not explored, potentially overlooking superior options. Thirdly, the study primarily focused on traditional and ensemble ML models, with limited investigation into deep learning techniques, which may offer enhanced capabilities for handling complex data. Furthermore, while class imbalance, a prevalent issue in cybersecurity data, was addressed through preprocessing, a more in-depth analysis is warranted. Lastly, although the study assessed model accuracy, the evaluation of computational efficiency, a crucial factor for real-time malware detection, was not extensively conducted.

For future work, researchers could test on more diverse datasets to see if the results hold up in different cases. Trying more advanced feature selection methods and deep learning models, like CNNs or transformers, might improve detection even more. Better ways to handle class imbalance, like cost-sensitive learning or undersampling techniques, could also help. Running these models in real-world environments would give a better idea of how they perform outside of controlled experiments. Another good direction would be studying how to defend against malware that tries to trick detection models. Finally, optimizing models to run faster on large datasets would make them more useful for real-time cybersecurity. By working on these areas, future research can help build better, more reliable malware detection systems.

## Electronic supplementary material

Below is the link to the electronic supplementary material.

Supplementary Material 1

## Data Availability

“The datasets used and/or analyzed during the current study available from the first author Rakibul Hasan (email: r.hasan.179@westcliff.edu) if anyone needs the data for this study.”
