# Supplementary material for: Enhancing malware detection with feature selection and scaling techniques using machine learning models
Source: Sci Rep. 2025 Mar 17;15:9122. doi: 10.1038/s41598-025-93447-x (PMC11914577; doi:10.1038/s41598-025-93447-x)
Supplement: Supplementary file 1 — Supplementary Material 1 [file 41598_2025_93447_MOESM1_ESM.docx]

import numpy as np

import pandas as pd

from sklearn.model_selection import train_test_split

from sklearn.preprocessing import StandardScaler, OneHotEncoder

from sklearn.ensemble import RandomForestClassifier, GradientBoostingClassifier

from sklearn.linear_model import LogisticRegression

from sklearn.svm import SVC

from sklearn.neighbors import KNeighborsClassifier

from sklearn.metrics import accuracy_score

import tensorflow as tf

from tensorflow.keras.models import Sequential

from tensorflow.keras.layers import Dense, Dropout

from lightgbm import LGBMClassifier

from sklearn.ensemble import ExtraTreesClassifier, BaggingClassifier

from sklearn.metrics import classification_report, confusion_matrix, accuracy_score

import seaborn as sns

import matplotlib.pyplot as plt

from tensorflow.keras.regularizers import l2

from tensorflow.keras.layers import BatchNormalization

from sklearn.tree import DecisionTreeClassifier

from sklearn.ensemble import ExtraTreesClassifier, BaggingClassifier

from sklearn.feature_selection import SelectKBest

from sklearn.feature_selection import chi2

from imblearn.over_sampling import SMOTE

from tensorflow.keras.utils import to_categorical

from scipy.io import arff

from sklearn.metrics import accuracy_score, precision_score, recall_score, f1_score, confusion_matrix, classification_report

from sklearn.preprocessing import LabelEncoder

from sklearn.feature_selection import RFE

from sklearn.linear_model import LogisticRegression

from sklearn.neighbors import NearestNeighbors

import matplotlib.pyplot as plt

from sklearn.metrics import confusion_matrix, classification_report

from sklearn.metrics import precision_recall_curve, roc_curve, auc

from sklearn.metrics import PrecisionRecallDisplay, RocCurveDisplay

from xgboost import XGBClassifier

from lightgbm import LGBMClassifier

from catboost import CatBoostClassifier

from sklearn.linear_model import LogisticRegression

from sklearn.naive_bayes import GaussianNB

from sklearn.svm import SVC

from sklearn.tree import DecisionTreeClassifier

from sklearn.ensemble import RandomForestClassifier

from sklearn.metrics import accuracy_score, classification_report

from sklearn.neighbors import KNeighborsClassifier

from sklearn.ensemble import GradientBoostingClassifier, AdaBoostClassifier

from sklearn.discriminant_analysis import QuadraticDiscriminantAnalysis, LinearDiscriminantAnalysis

from sklearn.linear_model import SGDClassifier, RidgeClassifier, LogisticRegression

from IPython.display import FileLink

from sklearn.feature_selection import RFECV

from sklearn.model_selection import StratifiedKFold

from sklearn.feature_selection import f_classif, SelectKBest, RFE

# Replace with your dataset path

data_path = '/kaggle/input/cic2022-ransomware/CICmalDroid_2020.csv'

# Load data

data = pd.read_csv(data_path)

# Separate features and target

X = data.drop(["Class"], axis=1)

y = data['Class']

print("Train features:", X.shape)

print("Train target:", y.shape)

# Label encoding for base classifiers

from sklearn.preprocessing import LabelEncoder

label_encoder = LabelEncoder()

y = label_encoder.fit_transform(y)

# Applying SMOTE

#smote = SMOTE()

#X, y = smote.fit_resample(X, y)

#print("Train features:", X.shape)

#print("Train target:", y.shape)

# Calculate the Pearson correlation matrix

corr_matrix = X.corr(method='kendall')

# Visualize the correlation matrix using a heatmap

plt.figure(figsize=(30, 30))

sns.heatmap(corr_matrix, annot=True, fmt='.2f', cmap='coolwarm', square=True)

plt.title('Feature Correlation Matrix')

plt.savefig('cor_heatmap1.png', format='png', dpi=300, bbox_inches='tight', pad_inches=0.1)

plt.show()

from sklearn.feature_selection import VarianceThreshold, SelectKBest, chi2, RFE

from sklearn.decomposition import PCA

from sklearn.ensemble import RandomForestClassifier

from sklearn.feature_selection import mutual_info_classif, SelectKBest, SelectFromModel

# 4. Principal Component Analysis

pca = PCA(n_components=80) # Reducing to 5 principal components

X = pca.fit_transform(X)

#X_test_pca = pca.transform(X_test_normalized)

# 5. Linear Discriminant Analysis (LDA)

lda = LDA(n_components=90) # LDA components depend on class count

X = lda.fit_transform(X)

from sklearn.preprocessing import StandardScaler, MinMaxScaler, RobustScaler, Normalizer

from sklearn.model_selection import train_test_split

# Assuming X and y are already defined

# Split the dataset into training and testing sets

X_train, X_test, y_train, y_test = train_test_split(X, y, test_size=0.2, random_state=42)

# Standard Scaling

standard_scaler = StandardScaler()

X_train_standard = standard_scaler.fit_transform(X_train)

X_test_standard = standard_scaler.transform(X_test)

# Min-Max Scaling

min_max_scaler = MinMaxScaler()

X_train_minmax = min_max_scaler.fit_transform(X_train)

X_test_minmax = min_max_scaler.transform(X_test)

# Robust Scaling

robust_scaler = RobustScaler()

X_train_robust = robust_scaler.fit_transform(X_train)

X_test_robust = robust_scaler.transform(X_test)

# Normalization

normalizer = Normalizer()

X_train_normalized = normalizer.fit_transform(X_train)

X_test_normalized = normalizer.transform(X_test)

from sklearn.preprocessing import StandardScaler, MinMaxScaler, RobustScaler, Normalizer

from sklearn.model_selection import train_test_split

# Standard Scaling

standard_scaler = StandardScaler()

#X = standard_scaler.fit_transform(X)

# Min-Max Scaling

min_max_scaler = MinMaxScaler()

#X = min_max_scaler.fit_transform(X)

# Robust Scaling

robust_scaler = RobustScaler()

#X = robust_scaler.fit_transform(X)

# Normalization

normalizer = Normalizer()

#X = normalizer.fit_transform(X)

# Split the dataset into training and testing sets

X_train, X_test, y_train, y_test = train_test_split(X, y, test_size=0.2, random_state=42)

import numpy as np

import seaborn as sns

import matplotlib.pyplot as plt

import tensorflow as tf

from tensorflow import keras

from tensorflow.keras.models import Sequential

from tensorflow.keras.layers import Dense, Dropout

from sklearn.linear_model import LogisticRegression, RidgeClassifier, SGDClassifier

from sklearn.naive_bayes import GaussianNB

from sklearn.svm import SVC

from sklearn.tree import DecisionTreeClassifier

from sklearn.ensemble import RandomForestClassifier, ExtraTreesClassifier, GradientBoostingClassifier, AdaBoostClassifier

from sklearn.neighbors import KNeighborsClassifier

from xgboost import XGBClassifier

from lightgbm import LGBMClassifier

from catboost import CatBoostClassifier

from sklearn.discriminant_analysis import LinearDiscriminantAnalysis, QuadraticDiscriminantAnalysis

from sklearn.metrics import accuracy_score, classification_report, confusion_matrix

# Scale up font sizes for plots

sns.set(font_scale=1.5)

# Initialize the models

logistic_regression_model = LogisticRegression(max_iter=1000)

naive_bayes_model = GaussianNB()

svm_model = SVC()

decision_tree_model = DecisionTreeClassifier()

random_forest_model = RandomForestClassifier()

et_model = ExtraTreesClassifier(n_estimators=100, random_state=42)

xgb_model = XGBClassifier(use_label_encoder=False, eval_metric='logloss')

lgbm_model = LGBMClassifier()

catboost_model = CatBoostClassifier(verbose=0) # verbose=0 to keep the output clean

knn_model = KNeighborsClassifier()

gbm_model = GradientBoostingClassifier()

adaboost_model = AdaBoostClassifier()

qda_model = QuadraticDiscriminantAnalysis()

lda_model = LinearDiscriminantAnalysis()

sgd_model = SGDClassifier(loss='log') # log loss makes it logistic regression

ridge_model = RidgeClassifier()

# Define ANN model

def build_ann(input_dim):

model = Sequential([

Dense(128, activation='relu', input_shape=(input_dim,)),

Dropout(0.3),

Dense(64, activation='relu'),

Dropout(0.3),

Dense(1, activation='sigmoid') # Change to softmax for multi-class classification

])

model.compile(optimizer='adam', loss='binary_crossentropy', metrics=['accuracy'])

return model

# List of traditional ML models

models = [

('LR', logistic_regression_model),

('NB', naive_bayes_model),

('SVM', svm_model),

('DT', decision_tree_model),

('ET', et_model),

('RF', random_forest_model),

('LGBM', lgbm_model),

('KNN', knn_model),

('GBM', gbm_model),

('LDA', lda_model),

('RC', ridge_model)

]

# Train and evaluate traditional ML models

for name, model in models:

model.fit(X_train, y_train) # Train model

y_pred = model.predict(X_test) # Predict on test data

accuracy = accuracy_score(y_test, y_pred)

print(f'{name} Accuracy: {accuracy * 100:.2f}%')

print(f'Classification Report for {name}:\n{classification_report(y_test, y_pred)}\n')

# Generate confusion matrix

cm = confusion_matrix(y_test, y_pred)

# Plot confusion matrix

plt.figure(figsize=(8, 6))

sns.heatmap(cm, annot=True, fmt='d', cmap='Blues', cbar=False, annot_kws={"size": 28})

plt.xlabel('Predicted labels', fontsize=20)

plt.ylabel('True labels', fontsize=20)

plt.xticks(fontsize=20)

plt.yticks(fontsize=20)

plt.savefig(f'cm_{name}.png', format='png', dpi=250, bbox_inches='tight', pad_inches=0.1)

#plt.show()

# Train and evaluate ANN separately

input_dim = X_train.shape[1]

ann_model = build_ann(input_dim)

# Train ANN

ann_model.fit(X_train, y_train, epochs=50, batch_size=32, validation_data=(X_test, y_test), verbose=1)

# Predict using ANN

y_pred_ann = (ann_model.predict(X_test) > 0.5).astype(int)

# ANN accuracy

ann_accuracy = accuracy_score(y_test, y_pred_ann)

print(f'ANN Accuracy: {ann_accuracy * 100:.2f}%')

print(f'Classification Report for ANN:\n{classification_report(y_test, y_pred_ann)}\n')

# Confusion matrix for ANN

cm_ann = confusion_matrix(y_test, y_pred_ann)

plt.figure(figsize=(8, 6))

sns.heatmap(cm_ann, annot=True, fmt='d', cmap='Blues', cbar=False, annot_kws={"size": 28})

plt.xlabel('Predicted labels', fontsize=20)

plt.ylabel('True labels', fontsize=20)

plt.xticks(fontsize=20)

plt.yticks(fontsize=20)

plt.savefig('cm_ANN.png', format='png', dpi=250, bbox_inches='tight', pad_inches=0.1)

#plt.show()

import pandas as pd

from sklearn.metrics import accuracy_score, precision_score, recall_score, f1_score, log_loss, roc_auc_score, cohen_kappa_score, classification_report

from sklearn.preprocessing import label_binarize

from sklearn.exceptions import UndefinedMetricWarning

import warnings

# Initialize a DataFrame to store the results

results = pd.DataFrame(columns=['Model', 'Accuracy', 'Precision', 'Recall', 'F1 Score', 'Log Loss', 'AUC', 'Cohen\'s Kappa'])

# Handle cases where a score cannot be computed

warnings.filterwarnings('ignore', category=UndefinedMetricWarning)

for name, model in models:

model.fit(X_train, y_train) # Fit model

y_pred = model.predict(X_test) # Predict on test set

y_proba = model.predict_proba(X_test)[:, 1] if hasattr(model, "predict_proba") else [0]*len(y_test)

# Compute metrics

accuracy = accuracy_score(y_test, y_pred)

precision = precision_score(y_test, y_pred, average='weighted')

recall = recall_score(y_test, y_pred, average='weighted')

f1 = f1_score(y_test, y_pred, average='weighted')

kappa = cohen_kappa_score(y_test, y_pred)

try:

ll = log_loss(y_test, y_proba)

# Handling multi-class for AUC if necessary

if len(np.unique(y_test)) > 2:

y_test_binarized = label_binarize(y_test, classes=np.unique(y_test))

auc = roc_auc_score(y_test_binarized, model.predict_proba(X_test), multi_class='ovr')

else:

auc = roc_auc_score(y_test, y_proba)

except ValueError:

ll = auc = 'NA' # Not applicable if predict_proba is not available or other issues

# Create a DataFrame for current model results and concatenate it

current_results = pd.DataFrame({

'Model': [name],

'Accuracy': [accuracy],

'Precision': [precision],

'Recall': [recall],

'F1 Score': [f1],

'Log Loss': [ll],

'AUC': [auc],

'Cohen\'s Kappa': [kappa]

})

results = pd.concat([results, current_results], ignore_index=True)

# Save results to an Excel file

results.to_excel('model_evaluation_pca_without.xlsx', index=False)

from sklearn.metrics import roc_curve, auc

import matplotlib.pyplot as plt

# Assuming 'models', 'X_train', 'y_train', 'X_test', and 'y_test' are defined

# Scale up the font sizes

sns.set(font_scale=1.5) # Adjust this value as needed

# Initialize lists to store false positive rates (fprs) and true positive rates (tprs) for each model

all_fprs = []

all_tprs = []

all_roc_aucs = []

# Loop through models, fit to training data, and evaluate

plt.figure(figsize=(8, 6))

for name, model in models:

model.fit(X_train, y_train) # Fit model to training data

if hasattr(model, "predict_proba"): # Check if the model has predict_proba method

y_pred_proba = model.predict_proba(X_test)[:, 1] # Predict probabilities for positive class

else:

y_pred_proba = model.decision_function(X_test) # Use decision function scores

fpr, tpr, _ = roc_curve(y_test, y_pred_proba) # Calculate ROC curve

roc_auc = auc(fpr, tpr) # Calculate AUC

# Plot ROC curve for each model

plt.plot(fpr, tpr, lw=2, label=f'{name} (AUC = {roc_auc:.2f})')

# Append fprs, tprs, and AUC scores to lists

all_fprs.append(fpr)

all_tprs.append(tpr)

all_roc_aucs.append(roc_auc)

# Plot random guessing line

plt.plot([0, 1], [0, 1], color='gray', linestyle='--')

# Set labels and title with larger font sizes

plt.xlabel('False Positive Rate', fontsize=18)

plt.ylabel('True Positive Rate', fontsize=18)

#plt.title('ROC Curves for All Models', fontsize=16)

plt.legend(loc='lower right', fontsize=16)

# Adjust layout

plt.tight_layout()

# Save the combined ROC curve plot with a higher DPI for better resolution

plt.savefig('combined_roc_curves_pca_without.png', format='png', dpi=300, bbox_inches='tight', pad_inches=0.1)

# Show the plot

plt.show()

# Optionally, print the AUC scores for each model with larger font size

#for name, roc_auc in zip([name for name, _ in models], all_roc_aucs):

# print(f'{name} AUC: {roc_auc:.2f}')

import matplotlib.pyplot as plt

import numpy as np

from sklearn.metrics import accuracy_score, precision_score, recall_score, f1_score

# Assuming 'models', 'X_train_normalized', 'y_train', 'X_test_normalized', and 'y_test' are defined

# Initialize lists to store evaluation metrics for each model

accuracy_scores = []

precision_scores = []

recall_scores = []

f1_scores = []

model_names = []

# Loop through models, fit to training data, and evaluate

for name, model in models:

model.fit(X_train, y_train) # Fit model to training data

y_pred = model.predict(X_test) # Predict on testing data

# Calculate evaluation metrics

accuracy = accuracy_score(y_test, y_pred)

precision = precision_score(y_test, y_pred)

recall = recall_score(y_test, y_pred)

f1 = f1_score(y_test, y_pred)

# Append evaluation metrics and model name to lists

accuracy_scores.append(accuracy)

precision_scores.append(precision)

recall_scores.append(recall)

f1_scores.append(f1)

model_names.append(name)

# Plot bar graphs for accuracy, precision, recall, and F1-score

fig, ax = plt.subplots(2, 2, figsize=(12, 10))

# Bar graph for accuracy

ax[0, 0].bar(model_names, accuracy_scores, color='b')

ax[0, 0].set_title('Accuracy', fontsize=14)

ax[0, 0].set_xlabel('Model', fontsize=14)

ax[0, 0].set_ylabel('Score', fontsize=14)

# Bar graph for precision

ax[0, 1].bar(model_names, precision_scores, color='g')

ax[0, 1].set_title('Precision', fontsize=14)

ax[0, 1].set_xlabel('Model', fontsize=14)

ax[0, 1].set_ylabel('Score', fontsize=14)

# Bar graph for recall

ax[1, 0].bar(model_names, recall_scores, color='r')

ax[1, 0].set_title('Recall', fontsize=14)

ax[1, 0].set_xlabel('Model', fontsize=14)

ax[1, 0].set_ylabel('Score', fontsize=14)

# Bar graph for F1-score

ax[1, 1].bar(model_names, f1_scores, color='purple')

ax[1, 1].set_title('F1-score', fontsize=14)

ax[1, 1].set_xlabel('Model', fontsize=14)

ax[1, 1].set_ylabel('Score', fontsize=14)

# Rotate x-axis labels for better readability and adjust font size

for axes in ax.flat:

axes.tick_params(axis='x', rotation=90, labelsize=14)

axes.tick_params(axis='y', labelsize=14)

# Adjust layout

plt.tight_layout()

plt.savefig('Res_without_pca.png', format='png', dpi=300, bbox_inches='tight', pad_inches=0.1)

# Show the plots

plt.show()
